# Type 3 Diabetes Mellitus: A Link Between Alzheimer's Disease and Type 2 Diabetes Mellitus

**DOI:** 10.7759/cureus.11703

**Published:** 2020-11-25

**Authors:** Omar Nisar, Hira Pervez, Bilvesh Mandalia, Muhammad Waqas, Harmandeep Kaur Sra

**Affiliations:** 1 Internal Medicine, Shalamar Medical and Dental College, Lahore, PAK; 2 Internal Medicine/Cardiology, Dow University of Health Sciences, Karachi, PAK; 3 Psychiatry, Sunshine Wellness Centre, Mumbai, IND; 4 Internal Medicine, Liaquat University of Medical and Health Sciences, Hyderabad, PAK; 5 Internal Medicine, Mercy Health, Toledo, USA

**Keywords:** dementia, alzheimer’s dementia, diabetes type 2, single nucleotide polymorphism, genetics, genes, type 3 diabetes, insulin resistance

## Abstract

Chronic diseases, as their name suggests, are progressive and can have overlapping features. Similar to this, Alzheimer's disease (AD) and diabetes mellitus (DM) fall into the category of chronic degenerative diseases. The global burden of these two ailments is manifold; hence, it seems important to view the pathophysiologic mechanisms of DM in the worsening of AD. Genetic as well as environmental factors are seen to play a role in the disease pathogenesis. Several genes, metabolic pathways, electrolytes, and dietary habits are seen to hasten brain atrophy. Lying behind this is the accumulation of amyloid precursor and tau - the misfolded proteins - within the brain substance. This mechanism is usually innate to AD itself, but the impact of insulin resistance, disturbing the homeostatic milieu, is seen as a powerful contributing factor aggravating the neuronal loss impairing an individual's memory. Since this neuronal loss is permanent, it may lead to complications as seen with AD. To reach a consensus, we conducted an electronic literature review search using different databases. This aided us in understanding the common aspects between AD and DM on genetic, molecular, cellular levels, as well as the impact of minerals and diet on the disease manifestation. We also found that despite exceptional work, additional efforts are needed to explore the relationship between the two entities. This will help physicians, researchers, and pharmaceuticals to frame remedies targeting the cause and avoid the progression of AD.

## Introduction and background

Alzheimer's disease (AD) is a chronic neurodegenerative disease leading to a progressive loss of neuronal tissue. Similar to AD, type 2 diabetes mellitus (T2DM) has a similar outlook. As the global burden of the two diseases is at a rapid incline, it deems essential to study the impact of T2DM on the progression and/or worsening of AD. Some pathophysiologic mechanisms are involved in the manifestation of the two diseases, yet an overview of the genetic complexities requires special attention [1]. Many researchers around the globe can signify many genes associated with single-nucleotide polymorphisms (SNP), various metabolic pathways, and alterations in the homeostatic milieu as a reason for the exacerbation in the brain atrophy in AD with T2DM, especially with insulin resistance [2]. The scope of our review is to look for an association on the genetic level of the two diseases.

A detailed literature search was conducted using "PubMed" and "Google Scholar" as databases. "Alzheimer's Disease", "Type 3 Diabetes Mellitus", "Diabetes Mellitus Type 2", "Genes", "Single Nucleotide Polymorphisms", "Dementia" were used as MeSH terms. All the searched and reviewed articles were principally peer-reviewed, were in English, and published between January 2010 and May 2020. The initial articles were used for reference citations to provide additional supporting evidence. All the abstracts and published literature were included, and a thorough screening was performed to exclude anything beyond the scope of the manuscript. AD due to any other cause, type 1 diabetes, dementia due to other causes, and so on were part of the exclusion criteria. Full-text articles were reviewed and screened for the authenticity of the manuscript.

## Review

AD, the most common cause of late-onset dementia, is a progressive degenerative disorder of the nervous system. It is known to be the sixth leading cause of death in the United States, affecting about 5.5 million Americans [1], with around 34 million people affected worldwide [2]. Since the disease is multifactorial, a combination of both genetic and environmental aspects increases an individual’s incidence to develop this ailment in life. A salient attribute of the disease is extensive neuronal loss due to tau and Amyloid beta (A-beta) protein accumulation [3]. However, the genetic component is known to cause early-onset familial AD, one of the rare forms. Genes involving the metabolism of A-beta polymer may spontaneously mutate, leading to the early disease manifestations. Among these, the most common ones are amyloid precursor protein (APP), presenilin1 (PSEN1), and presenilin2 (PSEN2). This leads to the accumulation of oligomers of A-beta in the memory zones of the brain, especially the hippocampus and the cortex [4]. T2DM, another chronic disease, presents either as an absence of insulin most likely due to beta-cell burnout and/or insulin resistance. This may occur due to defective signaling at the cellular level, which may contribute to pancreatic burnout and hence the loss of glycemic controls [5]. The mechanism by which T2DM may involve the body is manifold. It is known to be a risk factor for many conditions such as cardiovascular, cerebrovascular diseases, and associated neuropathies [1]. Since the brain utilizes insulin, any alteration in the normal milieu can have a significant impact on this organ. This leads to an over-activation of glycogen synthase kinase-3 (GSK-3), production and modification of tau proteins, and neurofibrillary degeneration [6]. A two-fold increase in the incidence of AD has been reported in patients with T2DM [7,8]. Insulin helps in the degradation, transportation, and modified deposition of A-beta out of the brain. However, as defective insulin signaling can hamper these processes, it leads to abnormal accumulation of these misfolded peptides within the brain [9]. Similar to AD, T2DM is a degenerative disease leading to cellular loss within the beta cells of the pancreas. Since there are many mechanisms by which T2DM may augment AD, our main focus is to highlight certain genetic co-relations between the two of them.

Common pathophysiology of diabetes mellitus and Alzheimer's disease

Apart from the natural course of the two diseases, aging remains a high risk factor as for many other ailments. The common pathophysiology shared by the two conditions, such as insulin resistance, inflammatory stress, and aggregation of amyloid, and yet not limited to the variable cognitive alterations make it even more necessary to understand which disease has more impact on the other. By far, insulin resistance with or without diabetes remains a significant risk factor for AD. This is also related to the receptor function, where insulin-like growth factor 1 (IGF1) resistance and insulin receptor substrate (IRS) 1 and 2 dysfunctions may be triggered by the A-beta oligomers and hormonal resistance, ultimately leading to notorious cognitive decline [10]. This raises a question of whether central insulin resistance is the culprit or peripheral resistance. Both the diseases are chronic, and justifying the impact of one disease process on the other is of main interest for researchers. Literature suggests that there are around 415 million adults with T2DM, which is expected to be 640 million by 2040. Likewise, there are around 40 million demented individuals, with the proportion increasing to 110 million by 2050 [7]. The duration for the presence of AD and the appearance of symptoms with or without co-existent T2DM may provide a clue to the pathophysiological connection between the two diseases. Owing to the progressive nature of AD, it can be divided into four different stages: a stage with undetectable pathology termed as pre-disease, a preclinical stage with pathological manifestation but without cognitive decline, a stage of pre-dementia with mild cognitive impairment, and the stage of full-blown dementia. The presence of diabetes before or during these stages can predict the role of insulin resistance as a culprit for either initiation or worsening the course of AD. It is also evident from data that peripheral insulin resistance can be a contributing factor in the development of insulin resistance in the brain, leading to a reduction in the glucose uptake and ultimately increasing A-beta levels [11]. The formation of neuritic plaques is hastened in the presence of hyperinsulinemia and hyperglycemia [12]. Another entity that is seen in the literature is that the ratio of cerebrospinal fluid to blood plasma concentrations of insulin is decreased in advanced stages of AD. This is also shown in patients who have a functional loss of the APOE-4 allele. The alterations in the mitochondrial machinery remain the main link between the two diseases [13]. As an anabolic hormone, insulin has a protective role against A-beta protein accumulation within the brain as it prevents increased oxidative stress. Hence, insulin resistance especially with T2DM may be a hidden risk factor for the progression of AD, increasing the susceptibility of the brain to A-beta proteins [14].

Structure of amyloid precursor protein

A-beta (1-42) is a soluble protein. A study conducted by Baram et al. showed that amylin (1-37) seeds onto A-beta (1-42) by oligomerization. This is the product of cross-seeding among several amyloidogenic proteins leading to the formation of amylin A-beta plaques [3,15]. These plaques then aggregate within the brain contributing to the etiology of AD. Since both the diseases are progressive and since A-beta protein and its aggregates are degenerative proteins that accumulate chronically, these mechanisms describe why it is necessary to explore the genetic correlation between the two diseases.

Genetic association between type 2 diabetes mellitus and Alzheimer's disease

A study by Hu et al. described in detail the approach by which a genetic link can be established between the two diseases. According to the authors, understanding the association analysis versus the causation analysis is the most important aspect to describe both the diseases. In addition to this, common paths of the two disease processes need to be evaluated. These may include DNA methylation, phenotypic variations, and gene expressions. The study concluded that around 759 genes were found related to a shared genetic locus, leading to the disease process, of which 5 genes were directly and 682 were indirectly related to T2DM and AD. As indicated in the study, the authors reviewed the causative aspect that led to the diseases. Several pathways were directly or indirectly connected to the disease process. Of note were 16 pathways that directly linked both the diseases together. One pathway that caught attention was the CREBBP, MAPK, and PI3K-AKT. The enzyme RNA polymerase is directly regulated by the cyclic adenosine monophosphate response element. This response element binds to its transcriptional factor, both of which play a role in the long term memory. However, disturbance in the insulin signaling adversely impacts the working memory in patients with T2DM. GRMD1B and RP1-111D6.3 were the two main expression genes. Six phenotypes were found directly connected to the disease pathophysiology. Methylation can lead to variable gene expressions in the POU3F2, KIF4B, and TMSL3 within the dopaminergic synapses and adenosine monophosphate-activated protein kinase (AMPK) pathways leading to the genetic connection of both the diseases [5]. AMPK is known to be an energy modulator; it can stimulate the uptake of glucose in skeletal muscles and the catabolic effect of fatty acids in the adipose tissues by insulin sensitization. However, the overactivity of AMPK can lead to brain damage - modification of tau protein and loss of axonal growth [16]. Kulas et al. in their study found APP in the pancreatic tissue specifically in the pancreatic beta cells [17]. As the neurons and the beta Langerhans cells have genotypic and phenotypic similarities, the genetic expression of either organ can impact the other. Several researchers used mouse models to verify the previous studies linking insulin resistance to rapid cognitive decline owing to the pathology of AD. Two insulin-resistant states were created in transgenic mice - high-fat diet feeding and the genetic disruption of the insulin receptor substrate - to look for APP aggregate formation. It was concluded that the diseases are not just linked genetically but that diet-inducing metabolic stress and inflammation can also cause amyloid pathology [18]. Not only causation analysis but also association analysis, especially the genetic association analyses, has proven the link between these two diseases. Since T2DM is multi-factorial, the exploration of SNPs can help understand the link between these two diseases [9]. A study conducted by Silver et al. identified genes such as AKT2, PIK3CB, IGF1R, PIK3CD, MTOR, IDE, AKT1S1, AKT1, which had a significant relation with the resistance of insulin on the brain, leading to cognitive impairment. These genes were also found to have SNPs. Related to this study Silver et al. linked genetic variations that can lead to brain atrophy due to insulin resistance [19].

Type 3 diabetes mellitus

The mechanism by which insulin promotes the entry of glucose in various cells in the body is well known. Resistance to insulin hampers this process, leading to T2DM with a hyperglycemic environment. But can the same pathological process lead to the progression of AD is less well understood. However, the term “type 3 DM” is invariably used in the insulin-resistant environment prevailing in the neuropathogenic condition of AD. It is also known that AD does not have hyperglycemia and that the brain tissue is independent of glucose entry using insulin [20].

The common role of calcium in Alzheimer's disease and diabetes mellitus

A normal homeostatic milieu can be disturbed by different mechanisms, leading to the development of prospective diseases. Similarly, both T2MD and AD have a common association with an imbalance in calcium homeostasis and disease progression. “Calcium hypothesis” has caught the eye of every researcher for two decades. The link between calcium imbalance and oxidative stress governs this well-established theory. On a molecular level in AD patients, any changes that lead to free radical production is truly the reason for a sustained elevation of calcium. This impacts metabolic processes within the mitochondria, leading to premature activation of oxygenases. It has been speculated that any alteration in calcium homeostasis can lead to oxidative stress on the brain and/or pancreas similar to any cell of the body [21]. An increase in the intracellular calcium in AD can cause abnormal activation of calcium-dependent and calcium/calmodulin-dependent protein kinases, impairing the neuronal synapses and eventually leading to neuronal loss with the formation of A-beta aggregates and hyperphosphorylation of tau protein. T2DM has a similar molecular environment in terms of intracellular calcium dysregulation, leading to an additive effect when combined with AD. Some researchers used mouse genotype models to illustrate the tau phosphorylation in a diabetic environment, and this was pertinent with the imbalance of calcium, leading to impairment in the signaling within the cellular machinery [21].

## Conclusions

Insulin resistance plays a cardinal role in the progression of AD. To understand this mechanism, a thorough understanding of the disease pathophysiology is required. As discussed in this review, this includes various environmental aspects such as dietary habits and the involvement of agents at the cellular level. Common genetic factors are seen to be involved in the worsening of AD in an environment of T2DM, with insulin resistance in particular. It also leads to uncontrolled inflammatory stress on the neuronal tissue, which can precipitate the formation of amyloid and tau proteins, thus worsening AD. These facts clarify the in-depth learning of AD pathophysiology in T2DM, which can assist in the prevention, therapeutic approach, and decreasing the morbidity associated with the two diseases.
